# CD3+ B-1a Cells as a Mediator of Disease Progression in Autoimmune-Prone Mice

**DOI:** 10.1155/2018/9289417

**Published:** 2018-12-23

**Authors:** Wakako Yamamoto, Hidemi Toyoda, Dong-qing Xu, Ryo Hanaki, Mari Morimoto, Daisuke Nakato, Takahiro Ito, Shotaro Iwamoto, Motoki Bonno, Shigeki Tanaka, Masahiro Hirayama

**Affiliations:** ^1^Department of Pediatrics, Mie University Graduate School of Medicine, 2-174 Edobashi, Tsu, Mie 514-8507, Japan; ^2^Department of Neonatology and Pediatrics, Mie Central Medical Center, 2158-5 Hisaimyojincho, Tsu, Mie 514-1101, Japan

## Abstract

B-1a cells are distinguishable from conventional B cells, which are designated B-2 cells, on the basis of their developmental origin, surface marker expression, and functions. In addition to the unique expression of the CD5 antigen, B-1a cells are characterized by the expression level of CD23. Although B-1a cells are considered to be independent of T cells and produce natural autoantibodies that induce the clinical manifestations of autoimmune diseases, there is much debate on the role of B-1a cells in the development of autoimmune diseases. We examined the involvement of B-1a cells in autoimmune-prone mice with the *lpr* gene. MRL/*lpr* and B6/*lpr* mice exhibited lupus and lymphoproliferative syndromes because of the massive accumulation of CD3+CD4-CD8-B220+ T cells. Interestingly, the B220+CD23-CD5+ (B-1a) cell population in the peripheral blood and peritoneal cavity increased with age and disease progression. Ninety percent of B-1a cells were CD3 positive (CD3+ B-1a cells) and did not produce tumor necrosis factor alpha, interferon gamma, or interleukin-10. To test the possible involvement of CD3+ B-1a cells in autoimmune disease, we tried to eliminate the peripheral cells by hypotonic shock through repeated intraperitoneal injections of distilled water. The fraction of peritoneal CD3+ B-1a cells decreased, and symptoms of the autoimmune disease were much milder in the distilled water-treated MRL/*lpr* mice. These results suggest that CD3+ B-1a cells could be mediators of disease progression in autoimmune-prone mice.

## 1. Introduction

Systemic lupus erythematosus (SLE) is an autoimmune disease characterized by variability in clinical manifestation and multiorgan involvement. The complete etiology of SLE is still unknown, with contributions from genetic, epigenetic, hormonal, and environmental factors that drive the breakdown of immune cell tolerance, immune attack on target tissues, and subsequent development of disease in susceptible individuals [1]. A hallmark of the disease is the production of autoantibodies, which are mainly directed against nuclear antigens such as double-stranded DNA (dsDNA) or RNA-containing proteins such as the Sm antigen or RNP [2]. An attack by these autoantibodies and immune cells results in the damage of multiple organs, such as the kidney, skin, joints, central nervous system, and vascular system. Although the production of anti-dsDNA antibodies is driven by CD4 T cells, SLE is not only characterized by the production of specific CD4 T cell-driven autoantibodies but also by polyclonal B cell activation and hypergammaglobulinemia [3].

B cells can function as antigen-presenting cells that stimulate autoreactive T cells by promoting an inflammatory microenvironment to regulate SLE [4]. Upon antigen stimulation, B cells coordinate with CD4+ T cells to form germinal centers in peripheral lymphoid tissues, such as the spleen, lymph nodes, and Peyer's patches. In patients with SLE, activated memory B cell subsets are correlated with disease activity, and proportions of CD24^high^CD38^high^ transitional B cells are higher in patients with SLE than in the control individuals [5, 6]. Furthermore, qualitative and quantitative modifications of the CD5+ B-1 cell subsets have been reported in patients with SLE [7].

In mice, mature B cells can be classified into three major subsets: (1) follicular B cells, also known as B-2 cells, located in lymphoid follicles; (2) marginal zone (MZ) B cells localized proximal to the marginal sinus of the spleen; and (3) B-1 cells, which are most abundant in the peritoneal and pleural cavities [8]. B-2 cells mount antibody responses in a T cell-dependent manner, whereas both MZ B cells and B-1 cells generate T cell-independent responses [8]. Depending on the presence or absence of surface CD5, a pan T cell marker, B-1 cells can be further subdivided into B-1a (CD5+) and B-1b (CD5-) populations [8, 9]. B-1a cells are involved in the innate immune system, which is able to sense pathogen-associated molecular patterns and initiate an immune response by the secretion of natural polyreactive antibodies, thus limiting bacterial spread before the induction of an adaptive immune reaction [10, 11]. The natural antibodies secreted by B-1a cells not only neutralize invading pathogens but also recognize and clear dying cells, leading to the suppression of uncontrolled inflammation and autoimmunity [8, 12].

In mouse models for SLE, an increase in the percentage of CD5+ B-1a cells is one of the most common features [13–15]. In fact, mice that lack natural antibodies are prone to accelerated development of IgG autoantibodies and more severe autoimmune diseases, presumably because antigens and inflammation associated with apoptotic cell debris stimulate B-2 cell responses when not properly cleared in a timely fashion [8, 16]. However, several findings have suggested that the role of B-1 cells in autoimmune pathogenesis, through the production of low-affinity antibodies, diminished negative regulation and recruitment to germinal center reactions, or production of interleukin- (IL-) 10 [10, 17, 18]. Therefore, the role of B-1a cells in autoimmune diseases is still unclear.

In the present study, the involvement of B-1a cells in lupus-prone mice was investigated. Our results demonstrated that the B-1a cell population in the peripheral blood and peritoneal cavity (PerC) increased with age and 90% of the B-1a cells were CD3+ (CD3+ B-1a cells). Elimination of the peritoneal B-1a cells by hypotonic shock with repeated intraperitoneal (i.p.) injections of distilled water (dH_2_O) resulted in a decrease in the number of peripheral CD3+ B-1a cells and milder symptoms of autoimmunity in the dH_2_O-treated lupus-prone mice. These results suggest that CD3+ B-1a cells could be mediators of disease progression in lupus-prone mice.

## 2. Materials and Methods

### 2.1. Animals

Six-week-old female C57BL/6 (B6), C57BL/6-*lpr*/*lpr* (B6/*lpr*), and MRL/MPJ-*lpr*/*lpr* (MRL/*lpr*) mice were purchased from Japan SLC (Shizuoka, Japan). All the animals were maintained in a humidity- and temperature-controlled laminar flow room. The animals were cared for and handled in accordance with the guidelines of the National Institutes of Health and Institute for Animal Experimentation of Mie University. All procedures and experiments were approved by the Animal Ethics Committee (Permission number 29-17), Mie University Graduate School of Medicine.

### 2.2. Clinical Symptoms

The mice were marked individually, checked every day for survival, and examined for physical signs of disease. Renal disease was evaluated on the basis of the development of albuminuria every week, as described previously [19]. Proteinuria was measured colorimetrically by using commercially available sticks (tetrabromophenol paper; Eiken Chemical Co., Tokyo, Japan) and fresh urine samples. This colorimetric assay, which is relatively specific for albumin, was graded from 0 to 4+, and the approximate protein concentrations were as follows: 0, 0 mg/dl; ±, 15 mg/dl; 1+, 30 mg/dl; 2+, 100 mg/dl; 3+, 300 mg/dl; and 4+, >1000 mg/dl. High-grade proteinuria was defined as higher than 2+ (100 mg/dl). Cervical, axillary, and inguinal lymph node hyperplasias, 5 mm or larger, were visually monitored every week, from 6 weeks of age.

### 2.3. B-1 Cell Depletion

B-1 cells were depleted using an adaptation of the protocol reported by Murakami et al. [20] and Peterson et al. [21], in which i.p. injection of dH_2_O results in the selective depletion of B-1 cells in the PerC. dH_2_O (Otsuka Pharmaceutical Co., Ltd., Tokushima, Japan) was injected every week into the PerC, and the dose was 1 ml from 6 to 8 weeks of age and 2 ml from 8 weeks until sacrifice. To determine the efficiency of depletion, cells were isolated from the PerC and flow cytometric analysis was performed.

### 2.4. Isolation and Detection of B-1a Cells

Peripheral blood was obtained by puncturing the retroorbital venous plexus of the eyes with a heparinized capillary tube. Peritoneal cells were obtained by injecting 8 ml of ice-cold phosphate-buffered saline (PBS; Nacalai Tesque Kyoto, Japan) into the PerC, gently massaging the cavity, and collecting lavage fluid containing peritoneal cells by using an 18-gauge needle [21, 22]. To detect B-1a cells, we used the following antibodies for the flow cytometry: fluorescein isothiocyanate; phycoerythrin; Alexa Fluor® 647; allophycocyanin (APC); and peridinin chlorophyll protein complex-conjugated CD45/B220, CD23, CD5, and CD3e (BD Pharmingen, Franklin Lakes, NJ; Bio-Rad, Hercules, CA; and BioLegend, San Diego, CA). The B-1a cells were defined as B220+CD23-CD5+ cells [23–25] and analyzed using the BD fluorescence-activated cell sorting FACS Canto II Flow Cytometer (BD Bioscience, Franklin Lakes, NJ) with FACSDiva software (BD Bioscience).

### 2.5. Intracellular Cytokine Staining

Mononuclear cells were isolated using Histopaque®-1077 (Sigma-Aldrich) from the peripheral blood and PerC cells. The isolated cells were resuspended (1 × 10^6^ cells/ml) in complete medium (RPMI 1640 media (Wako Pure Chemical Industries, Osaka, Japan) containing 10% fetal bovine serum (FBS; Gibco, Waltham, MA), 200 *μ*g/ml penicillin, 200 U/ml streptomycin (Sigma-Aldrich), 4 mM L-glutamine, and 5 × 10^−5^ M 2-mercaptoethanol (Sigma-Aldrich)) with 10 *μ*g/ml of lipopolysaccharide (LPS; Sigma-Aldrich), 50 ng/ml of phorbol myristate acetate (PMA; Sigma-Aldrich), 500 ng/ml of ionomycin (Sigma-Aldrich), and 2 *μ*M monensin (eBioscience, San Diego, CA) and incubated at 37°C in 5% CO_2_ atmosphere for 5 h, as described previously [26, 27]. After cell-surface staining with CD3, CD5, and B220, as described above, the cells were fixed and permeabilized using IntraStain (Dako, Santa Clara, CA), according to the manufacturer's instructions. The permeabilized cells were stained with APC-conjugated mouse anti-tumor necrosis factor alpha (TNF*α*; eBioscience), interferon gamma (IFN*γ*; eBioscience), and IL-10 (eBioscience).

### 2.6. Statistical Analysis

The data were expressed as mean ± SEM values for each group. The statistical analysis was performed using GraphPad Prism version 7.03 for Windows (GraphPad Software, San Diego CA). Normal distribution of data was tested using the Shapiro–Wilk omnibus normality test. If two independent groups were not normally distributed and could not be transformed to a normal distribution by logarithmic transformation, we used the nonparametric Mann–Whitney test. If two independent normally distributed groups were compared, we used an unpaired *t*-test. To assess differences between multiple groups, nonparametric one-way analysis of variance on ranks (Kruskal–Wallis) test was used with Dunn's post hoc evaluation. A *p* value < 0.05 was considered statistically significant.

## 3. Results

### 3.1. Clinical Symptoms of Autoimmunity

**Figure 1:**
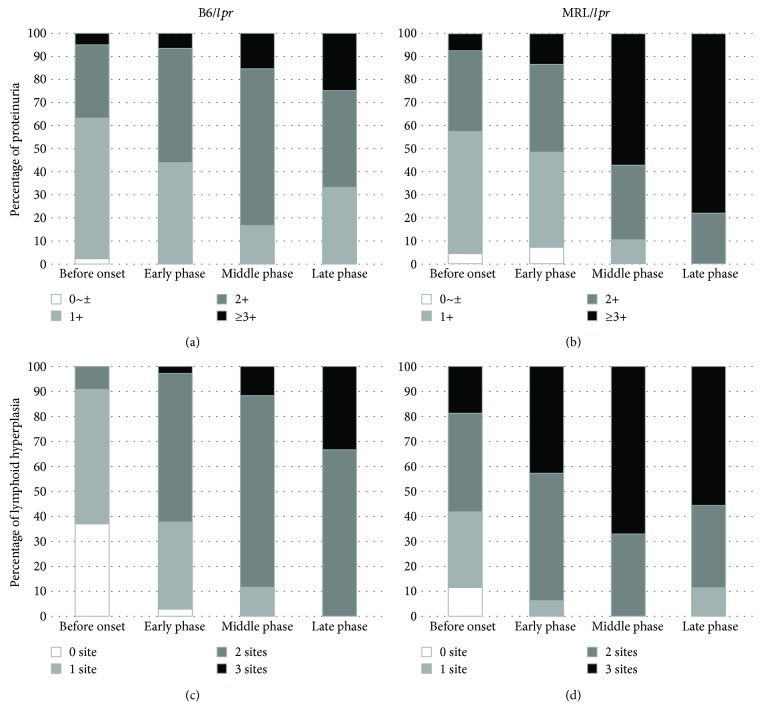
Cumulative prevalence of proteinuria and lymphoid hyperplasia. Urine protein and lymphadenopathy were monitored every week, starting at 6 weeks of age. Urine protein measured using tetrabromophenol paper over time in the B6/*lpr* (a) and MRL/*lpr* (b) mice. Cervical, axillary, and inguinal lymph node hyperplasias, 5 mm or larger, were monitored visually in the B6/*lpr* (c) and MRL/*lpr* (d) mice.

Clinical symptoms such as proteinuria and lymphoid hyperplasia were monitored in the B6/*lpr* and MRL/*lpr* mice (Figure 1). According to the progression of autoimmune symptoms, the disease was divided into four stages: before the onset of symptoms (6–9 weeks after birth), early phase after the onset of symptoms (10–14 weeks), middle phase after the onset of symptoms (15–29 weeks), and late phase after the onset of symptoms (30–34 weeks). As shown in Figure 1, the prevalence of proteinuria (greater than 2+) was 50% at the age of 11 weeks in the B6/*lpr* mice (Figure 1(a)) and 13 weeks in the MRL/*lpr* mice (Figure 1(b)). Lymphoid hyperplasia at more than two sites was detected at the age of 13 weeks in the B6/*lpr* mice (Figure 1(c)) and 8 weeks in the MRL/*lpr* mice (Figure 1(d)). The MRL/*lpr* mice showed a rapidly progressive increase in proteinuria and lymphoid hyperplasia when compared with the B6/*lpr* mice (Figure 1). The MRL/*lpr* mice died of the disease as early as 18 weeks. In the MRL/*lpr* mice, lymphoid hyperplasia seemed to improve in the late phase of the disease because of the poor survival of the mice.

### 3.2. Increased Peripheral B-1a Cells in the B6/*lpr* and MRL/*lpr* Mice

**Figure 2:**
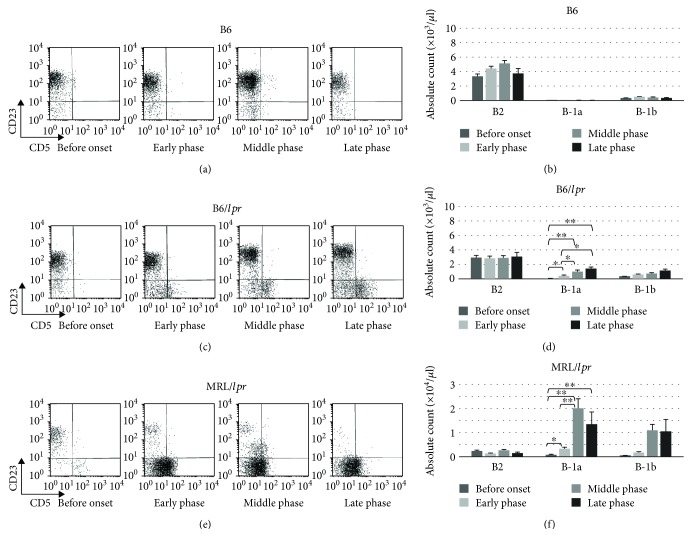
Cell-surface markers of B cells. Peripheral blood cells obtained from the B6 mice (*n* = 32) were stained and analyzed with flow cytometry. Representative flow cytometry plots (a) and absolute count of B-1a, B-1b, and B-2 cells (b) are shown. Peripheral blood cells obtained from the B6/*lpr* mice (*n* = 35) were stained and analyzed with flow cytometry. Representative flow cytometry plots (c) and absolute count of B-1a, B-1b, and B-2 cells (d) are shown. Peripheral blood cells obtained from the MRL/*lpr* mice (*n* = 27) were stained and analyzed with flow cytometry. Representative flow cytometry plots (e) and absolute count of B-1a, B-1b, and B-2 cells (f) are shown.

The number and frequency of B220+CD23-CD5+ B (B-1a), B220+CD23-CD5- B (B-1b), and B220+CD23+CD5- B (B-2) cells in the peripheral blood were sequentially investigated in the B6 (Figures 2(a) and 2(b)), B6/*lpr* (Figures 2(c) and 2(d)), and MRL/*lpr* mice (Figures 2(e) and 2(f)). A significant increase in the number and proportion of B-1a cells was observed with disease progression in the B6/*lpr* (Figures 2(c) and 2(d)) and MRL/*lpr* (Figures 2(e) and 2(f)) mice, but not in the B6 mice (Figures 2(a) and 2(b)). Although the MRL/*lpr* mice showed a rapid and early increase in B-1a cells in the peripheral blood (Figures 2(e) and 2(f)), the increase in B-1a cells was delayed and minimal in the B6/*lpr* mice (Figures 2(c) and 2(d)).

### 3.3. Increased Peripheral CD3+CD4-CD8-B220+ T Cells in the B6/*lpr* and MRL/*lpr* Mice

**Figure 3:**
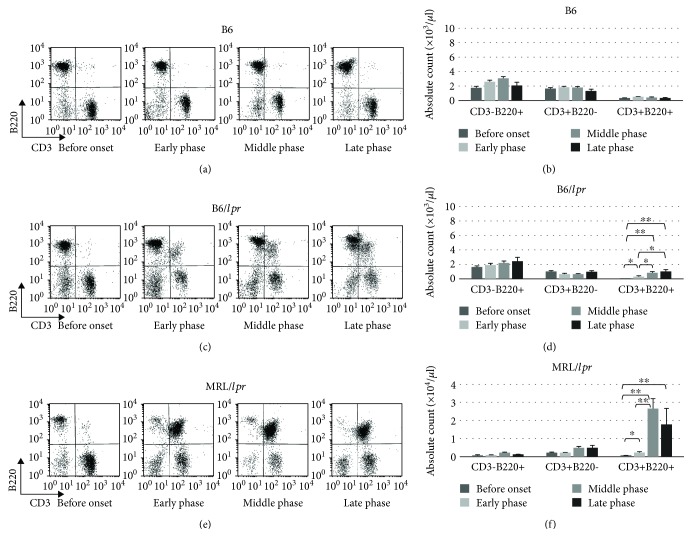
Expression of CD3 and B220 in peripheral blood. Peripheral blood cells obtained from the B6 mice (*n* = 32) were stained and analyzed with flow cytometry. Representative flow cytometry plots (a) and absolute count of CD3-B220+, CD3+B220-, and CD3+B220+ cells (b) are shown. Peripheral blood cells obtained from the B6/*lpr* mice (*n* = 35) were stained and analyzed with flow cytometry. Representative flow cytometry plots (c) and absolute count of CD3-B220+, CD3+B220-, and CD3+B220+ cells (d) are shown. Peripheral blood cells obtained from the MRL/*lpr* mice (*n* = 27) were stained and analyzed with flow cytometry. Representative flow cytometry plots (e) and absolute count of CD3-B220+, CD3+B220-, and CD3+B220+ cells (f) are shown.

Because accumulation of CD3+CD4-CD8-B220+ T cells plays a critical role in autoimmunity in lupus-prone mice [28, 29], the percentage and absolute count of CD3+CD4-CD8-B220+ T cells in the peripheral blood were examined (Figure 3). With disease progression, the population of CD3+CD4-CD8-B220+ T cells increased in the B6/*lpr* (Figures 3(c) and 3(d)) and MRL/*lpr* (Figures 3(e) and 3(f)) mice, but not in the B6 mice (Figures 3(a) and 3(b)). A massive proliferation of CD3+CD4-CD8-B220+ T cells was observed in the MRL/*lpr* mice when compared with the B6/*lpr* mice. Most of the CD3+B220+ cells were CD4-CD8- (data not shown).

### 3.4. Immunological Characteristics and Distribution of B-1a Cells in the B6/*lpr* and MRL/*lpr* Mice

**Figure 4:**
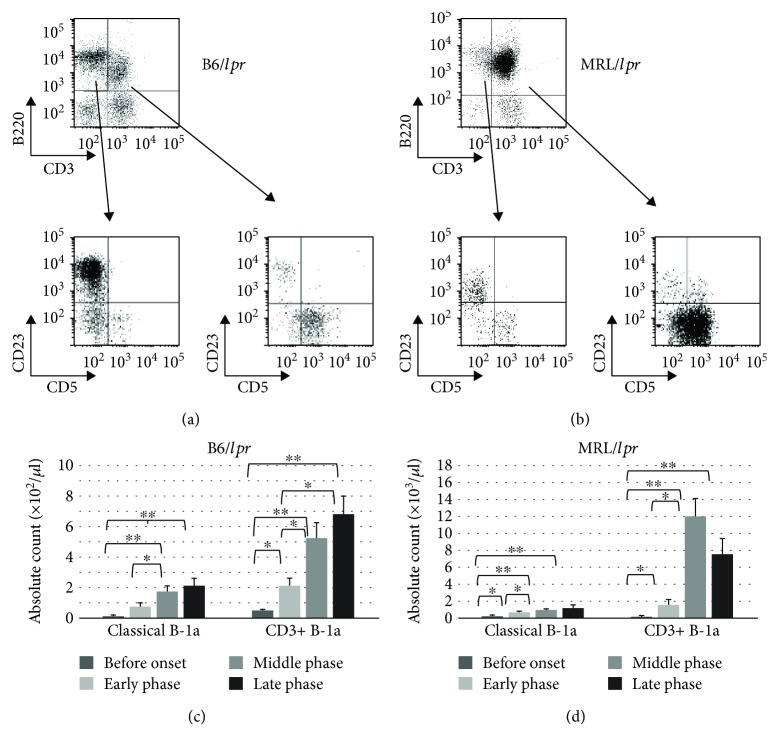
Detection of B-1a cells in the CD3+CD4-CD8-B220+ population. Peripheral blood cells obtained from the B6/*lpr* mice (*n* = 18) were stained with CD3, B220, and CD5 antibodies and analyzed using flow cytometry. Representative flow cytometry plots (a) and absolute count of classical B-1a cells and CD3+ B-1a cells (c) are shown. Peripheral blood cells obtained from the MRL/*lpr* mice (*n* = 21) were stained with CD3, B220, and CD5 antibodies and analyzed using flow cytometry. Representative flow cytometry plots (b) and absolute count of classical B-1a cells and CD3+ B-1a cells (d) are shown.

**Figure 5:**
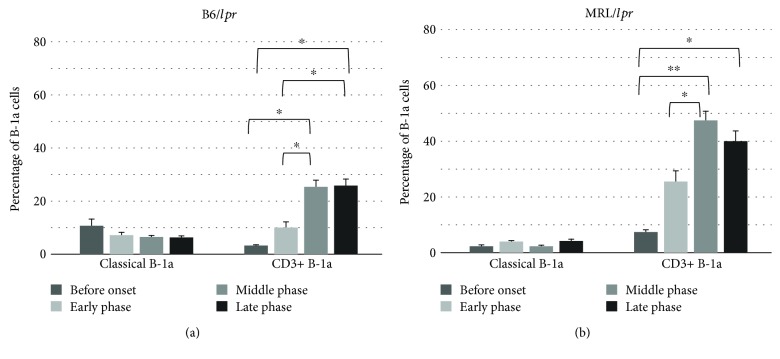
The relative percentage of classical B-1a and CD3+ B-1a cells in the peritoneal cavity. Peritoneal mononuclear cells obtained from the B6/*lpr* (*n* = 17) (a) and MRL/*lpr* (*n* = 16) (b) mice were stained with CD3, B220, CD5, and CD23 antibodies and analyzed using flow cytometry.

The B220+ cells in the B6/*lpr* and MRL/*lpr* mice could be divided into two main subsets, CD3-B220+ and CD3+B220+ cells, by CD3 intensity in the B6/*lpr* (Figure 4(a), upper panel) and MRL/*lpr* (Figure 4(b), upper panel) mice. CD5 intensity and CD23 surface expression defined three discrete subpopulations (B-1a, B-1b, and B-2) of CD3-B220+ and CD3+B220+ cells in the B6/*lpr* (Figure 4(a), lower panels) and MRL/*lpr* (Figure 4(b), lower panels) mice. Therefore, B-1a cells in lupus-prone mice consist of two principal subsets with CD3 surface expression, CD3+CD4-CD8-B220+CD23-CD5+ cells (CD3+ B-1a cells) and CD3-CD4-CD8-B220+CD23-CD5+ cells (classical B-1a cells). Both CD3+ B-1a and classical B-1a cells in the peripheral blood increased with age in B6/*lpr* (Figure 4(c)) and MRL/*lpr* (Figure 4(d)) mice. A massive accumulation of CD3+ B-1a cells was observed in the MRL/*lpr* mice (Figure 4(d)). Since B-1a cells are predominantly localized in the PerC, B-1a subsets in the PerC were characterized sequentially. As shown in Figure 5, the frequency of CD3+ B-1a cells increased with age in both B6/*lpr* (Figure 5(a)) and MRL/*lpr* (Figure 5(b)) mice. However, the frequency of classical B-1a cells in the PerC was not significantly affected by age and disease progression. A massive accumulation of CD3+ B-1a cells was observed in the MRL/*lpr* mice (Figure 5(b)) when compared with the B6/*lpr* mice (Figure 5(a)).

### 3.5. Cytokine Production of B Cells, T Cells, and CD3+B220+ Cells

**Figure 6:**
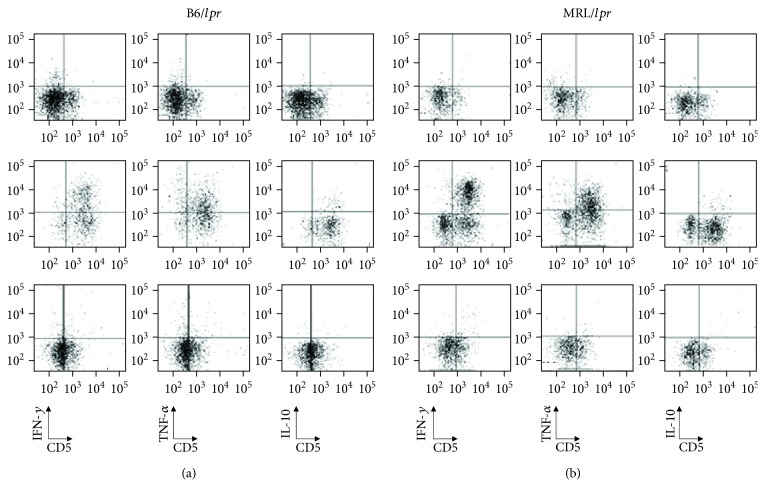
Intracellular staining for the detection of IFN*γ*, TNF*α*, and IL-10. Mononuclear cells isolated from the peripheral blood were cultured with LPS (10 *μ*g/ml), PMA (50 ng/ml), ionomycin (500 ng/ml), and monensin (2 *μ*M) for 5 h. After culture, the cells were stained with appropriate fluorescence antibodies to detect cell-surface markers, fixed, and permeabilized. The cells were also stained intracellularly with APC-conjugated anti-IFN*γ*, anti-TNF*α*, and anti-IL-10. After washing, the cells were immediately subjected to flow cytometric analysis. (a) Representative results of the flow cytometry of the B6/*lpr* mice showing intracellular staining of IFN*γ* (left column), TNF*α* (middle column), and IL-10 (right column) of B cells (upper line), T cells (middle line), and CD3+B220+ cells (lower line). (b) Representative results of flow cytometry of the MRL/*lpr* mice showing intracellular staining of IFN*γ* (left column), TNF*α* (middle column), and IL-10 (right column) of B cells (upper line), T cells (middle line), and CD3+B220+ cells (lower line).

Previous studies have suggested that B-1a cells are similar to regulatory B cells (Bregs), which possess the capacity to downregulate immune responses via the secretion of IL-10 [30]. To investigate whether B-1a cells in the peripheral blood produce IL-10, peripheral mononuclear cells were stimulated with LPS and analyzed for their potential capacity to produce cytokines, such as IL-10, IFN*γ*, and TNF*α*, in the B6/*lpr* (Figure 6(a)) and MRL/*lpr* (Figure 6(b)) mice. The peripheral B cells did not possess the potential capacity to produce IL-10, IFN*γ*, or TNF*α* in the B6/*lpr* (Figure 6(a), upper panels) and MRL/*lpr* (Figure 6(b), upper panels) mice. The LPS treatment did not increase the number of IL-10-producing peripheral T cells, but the stimulation did significantly alter the produced quantities of IFN*γ* and TNF*α* in the B6/*lpr* (Figure 6(a), middle panels) and MRL/*lpr* (Figure 6(b), middle panels) mice. The peripheral CD3+B220+ cells, including CD3+ B-1a cells, did not possess the potential capacity to produce IL-10, IFN*γ*, or TNF*α* in the B6/*lpr* (Figure 6(a), lower panels) and MRL/*lpr* (Figure 6(b), lower panels) mice.

### 3.6. Efficacy of B-1 Cell Depletion by Hypotonic Shock

**Figure 7:**
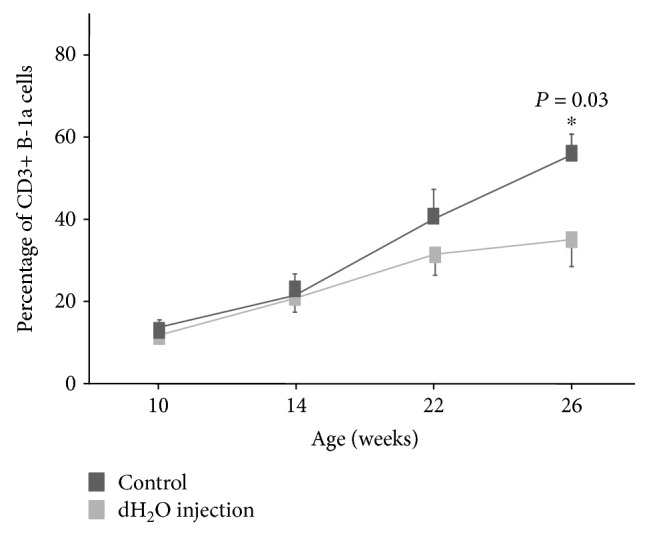
Depletion of CD3+ B-1 cells by repeated intraperitoneal injections of distilled water. Flow cytometric analysis of cells from the peripheral blood by using antibodies against CD3, B220, CD5, and CD23 was used to assess the depletion of CD3+ B-1a cells. The MRL/*lpr* mice, into which 1 ml of dH_2_O had been injected weekly from 6 weeks of age (dH_2_O injection; *n* = 5), showed a significant reduction in the frequency of CD3+ B-1a cells when compared with the control mice (control; *n* = 8).

Murakami et al. [20] and Peterson et al. [21] have reported that i.p. injection of dH_2_O resulted in a reduction of B-1 cells. Therefore, we evaluated the effect of the elimination of B-1 cells on the development of autoimmune symptoms in the lupus-prone mice. The frequency of classical B-1a cells in the PerC was 2% in the dH_2_O-treated MRL/*lpr* mice when compared with 4% in the control MRL/*lpr* mice (data not shown). Since i.p. dH_2_O treatment specifically eliminates B-1 cells, we examined whether the treatment also suppresses the proliferation of peripheral CD3+ B-1a cells. Water injection decreased the frequency of CD3+ B-1a cells, and the efficiency of depletion in the peripheral blood was 37.3% (Figure 7). Furthermore, the dH_2_O-treated MRL/*lpr* mice showed milder clinical signs, such as proteinuria and lymphoid hyperplasia, than the control mice (data not shown).

## 4. Discussion

The aim of the current study was to examine the potential functions of B-1a cells. Our investigations show that B-1a cells in lupus-prone mice can be subdivided into CD3- B-1a (classical B-1a) and CD3+ B-1a cells, and CD3+ B-1a cells are mediators of disease progression in the lupus-prone mice. The recently recognized importance of B cells in SLE raises the question as to whether those expressing CD5 predominate over the remaining B cells in the pathophysiology of this disease [7]. Although autoantibody production has been originally ascribed to B-1a cells, high-affinity autoantibodies have been established to be derived from B-2 cells [7, 11, 15, 17, 18, 31]. Therefore, B-1a cells have been considered to play a paradoxical role in preventing, rather than inducing, autoimmunity [7, 11, 15, 17, 18, 31]. A large increase in the number and proportion of B-1a cells in the peripheral blood and PerC represents a consistent phenotype in MRL/*lpr* and B6/*lpr* mice. Interestingly, more than 80% of the peripheral B-1a cells were CD3+CD4-CD8- in the B6/*lpr* mice, and more than 90% of the peripheral B-1a cells were CD3+CD4-CD8- in the MRL/*lpr* mice. Therefore, CD3+ B-1a and CD3+CD4-CD8-B220+ cells seem to be the exact same cells. Considering that the accumulation of CD3+CD4-CD8-B220+ cells plays a critical role in autoimmunity in lupus-prone mice [28, 29], the number and frequency of CD3+ B-1a cells could be contributing to the disease progression. The Shc family protein adaptor Rai is expressed in T and B lymphocytes, and acts as a negative regulator of lymphocyte survival and activation [32, 33]. Loss of this protein results in breaking of immunological tolerance and development of systemic autoimmunity in mice models [32]. T cells from SLE patients were found to have a defect in Rai expression [33]. Therefore, it is important to examine the expression of Rai in lymphocytes obtained from MRL/*lpr* and B6/*lpr* mice.

We have been using two autoimmune-prone strains of mice—MRL/*lpr* and B6/*lpr*— to investigate the potential functions of B-1a cells. Although these strains carry a defective mutation in the Fas gene denoted as *lpr* (for lymphoproliferation), onset and severity of symptoms were different. MRL/*lpr* mice develop severe early onset autoimmune disease characterized by massive lymphoadenopathy, abundant circulating autoantibodies, and fatal glomerulonephritis [34]. On the other hand, B6/*lpr* mice display delayed and minimal lupus nephritis [35, 36]. The observations in the present study are consistent with the notion that onset and severity of the *lpr*-induced phenotypes depend on the genetic background of *lpr* [34–36].

Among the B cell subsets, B-1a cells were first identified to have the ability to produce IL-10 [37, 38]. B-1a cells can spontaneously secrete IL-10, and the production of IL-10 can increase in response to the stimulation [37, 38]. A specialized population of IL-10-producing B cells has been characterized with regulatory function [39], and B-1a cells have been regarded to have regulatory function [30, 40]. However, in our study, peripheral B cells did not possess the potential capacity to produce IL-10 in the B6/*lpr* and MRL/*lpr* mice. A higher percentage of PerC B cells possess the potential capacity to produce IL-10, when compared with splenic B cells, after stimulation with *α*CD40, IL-21, or *α*CD40 in combination with 5 h of LPS [30]. Although peripheral B cells were stimulated with LPS for 5 h and IL-10-producing B cells were analyzed in our experiments, PerC B cells may be used and stimulated with not only LPS but also *α*CD40. Since the produced quantities of IL-10 were significantly increased by LPS treatment, *α*CD40 + LPS, or *α*CD40 + 5 h LPS [30], IL-10 secretion into the supernatant may be analyzed using the enzyme-linked immunosorbent assay in our experiments.

Expansion of the CD3+ B-1a cell component is one of the most characteristic phenotypes in lupus-prone mice. However, it is unclear whether CD3+ B-1a cells induce or regulate the clinical manifestations of the autoimmune disease. The MRL/*lpr* and B6/*lpr* mice exhibited lupus and lymphoproliferative syndromes because of the massive accumulation of CD3+CD4-CD8-B220+ cells, which are identical to CD3+ B-1a cells. Although B-1a cells are associated with the regulation of autoimmune disease through the secretion of anti-inflammatory cytokines [30], the CD3+ B-1a cells did not secrete IL-10. These results suggest that CD3+ B-1a cells contribute to lupus pathogenesis rather than disease suppression.

I.p. injection of dH_2_O resulted in a dramatic reduction of B cells, T cells, and macrophages in the PerC [20, 21]. Although the initial killing was nonspecific, the long-lasting depletion was specific to B-1 cells because they are the only cells that depend on self-renewal within the PerC for replenishment [21]. The efficiency of CD3+ B-1a cell depletion in the peripheral blood was 37.3% in our study. The severity of autoimmune symptoms decreased in the dH_2_O-treated MRL/*lpr* mice, but the effect was relatively mild when compared with previous studies [20, 21]. Several possibilities could explain why the depletion of CD3+ B-1a cells resulted in such a modest alteration in the clinical outcomes in the MRL/*lpr* mice. Our results for the effects of B-1 cell depletion (37.3%) differ from those reported by Murakami et al. [20] in New Zealand Black × New Zealand White F1 mice (87%) and those reported by Peterson et al. [21] in A.SW (*H-2^s^-T18^b^-/SnJ*) mice (70%). The mild effect could be due to the incomplete elimination of CD3+ B-1a cells, which are found predominantly in the PerC and peripheral blood but are also present in lymphoid organs (data not shown). Therefore, CD3+ B-1a cells outside the PerC and peripheral blood could contribute to the pathogenesis of proteinuria and lymphadenopathy. The mild effect could be also due to slight differences in the depletion protocol because the weekly i.p. injections were administered to the mice in our study from 6 weeks of age, whereas Murakami et al. [20] continued the i.p. water injections every 7 days for the neonate mice to eliminate the peritoneal cells. Rituximab, a chimeric anti-CD20 monoclonal antibody, has been used with success in recalcitrant lupus manifestations [41]. Since B-1a cells express CD20, rituximab used in clinic may alter B-1a cells.

In conclusion, B-1a cells in lupus-prone mice can be subdivided into CD3- B-1a and CD3+ B-1a cells, and CD3+ B-1a cells could be mediators of disease progression in the mice. Although studies on B-1a cells are premature in patients with SLE, specific elimination of B-1a cells may be useful for therapy, as shown in the present study.

## Data Availability

The data used to support the findings of this study are available from the corresponding author upon request.
